# Therapeutic targeting of PLK1 in *TERT* promoter‐mutant hepatocellular carcinoma

**DOI:** 10.1002/ctm2.1703

**Published:** 2024-05-20

**Authors:** Qin Tang, Guanghui Hu, Ye Sang, Yulu Chen, Guangyan Wei, Meiyan Zhu, Mengke Chen, Shiyong Li, Rengyun Liu, Zhenwei Peng

**Affiliations:** ^1^ Department of Radiation Oncology The First Affiliated Hospital Sun Yat‐sen University Guangzhou China; ^2^ Institute of Precision Medicine The First Affiliated Hospital Sun Yat‐sen University Guangzhou China; ^3^ Cancer Center The First Affiliated Hospital Sun Yat‐sen University Guangzhou China

**Keywords:** hepatocellular carcinoma, PLK1, Smad3, TERT promoter mutation

## Abstract

**Background:**

Hotspot mutations in the promoter of *telomerase reverse transcriptase* (*TERT*) gene are the most common genetic variants in hepatocellular carcinoma (HCC) and associated with poor prognosis of the disease. However, no drug was currently approved for treating *TERT* promoter mutation positive HCC patients. Here, we aim to explore the potential therapeutic strategy for targeting *TERT* promoter mutation in HCC.

**Methods:**

The Liver Cancer Model Repository database was used for screening potential drugs to selectively suppress the growth of *TERT* promoter mutant HCC cells. RNA‐seq, CRISPR‐Cas9 technology and siRNA transfection were performed for mechanistic studies. Cell counting kit‐8 (CCK8) assay and the xenograft tumour models were used for cell growth detection in vitro and in vivo, respectively. Cell apoptosis and cell cycle arrest were analysed by Annexin V‐FITC staining and/or propidium iodide staining.

**Results:**

PLK1 inhibitors were remarkably more sensitive to HCC cells harbouring *TERT* promoter mutation than wild‐type cells in vitro and in vivo, which were diminished after *TERT* promoter mutation was edited to the wild‐type nucleotide. Comparing the HCC cells with wild‐type promoter of *TERT*, PLK1 inhibitors specifically downregulated Smad3 to regulate TERT for inducing apoptosis and G2/M arrest in *TERT* mutant HCC cells. Moreover, knockout of Smad3 counteracted the effects of PLK1 inhibitors in *TERT* mutant HCC cells. Finally, a cooperative effect of PLK1 and Smad3 inhibition was observed in *TERT* mutant cells.

**Conclusions:**

PLK1 inhibition selectively suppressed the growth of *TERT* mutant HCC cells through Smad3, thus contributed to discover a novel therapeutic strategy to treat HCC patients harbouring *TERT* promoter mutations.

**Key points:**

- *TERT* promoter mutation confers sensitivity to PLK1 inhibitors in HCC.
- The selective growth inhibition of *TERT* mutant HCC cells induced by PLK1 inhibitor was mediated by Smad3.
- Combined inhibition of PLK1 and Smad3 showed a cooperative anti‐tumor effect in *TERT* mutant HCC cells.

## Graphical abstract

1. PLK1 inhibitor BI2536 preferred to suppress the growth of HCC cells harboring *TERT* promoter mutation.
2. PLK1 inhibition induced cell cycle arrest and apoptosis in TERT mutant HCC cells.
3. The selective inhibitory effects of PLK1 inhibitor on *TERT* mutant HCC cells was mediated by Smad3.

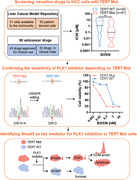

## 1 INTRODUCTION

Hepatocellular carcinoma (HCC) is an increasing threat for human health with high incidence and mortality rates, especially in China.^1^ China is the region of the highest incidence of primary HCC, and its incidence and mortality respectively account for 45.3% and 47.1% of the global cases.^1^ Although recently more and more therapies have been applied for HCC therapy,^2^, ^3^, ^4^ a majority of them are only contributed to limited survival benefit. Thus, exploring novel targets and powerful drugs for HCC therapy is urgent.

In the past two decades, a number of novel driver genes were identified in HCC,^5^, ^6^, ^7^ especially genetic mutation associated with hepatocarcinogenesis and progression. Among them, mutations in the core promoter region of telomerase reverse transcriptase (*TERT*) were frequently observed in 30−60% of HCC patients.^8^, ^9^ TERT is mainly responsible for telomerase reactivation and preventing telomere shortening.^10^, ^11^ Telomerase reactivation is a key event in HCC initiation and has been observed in more than 90% of HCC.^10^ The most prevalent mechanism for telomerase reactivation is *TERT* promoter mutations which reactivates *TERT* expression by creating a de novo binding site (TTCCGG) for E26 transformation‐specific (ETS) transcription factors, and thereby reactivates telomerase and maintains telomere length in the progression of HCC.^12^, ^13^ Although there are two hot spot *TERT* promoter mutations (G228A at −124 bp and G250A at −146 bp upstream the translation start site) in human cancers,^14^, ^15^ G228A occurs dominantly in HCC.^16^

Clinically, *TERT* promoter mutations are related with poor clinical outcomes in multiple types of human cancer, including HCC.^17^, ^18^, ^19^, ^20^ To date, recent studies revealed that GABPB1L and TRIM28 had the potential to be the therapeutic target for *TERT* promoter mutation‐positive patients with glioblastoma and bladder cancer, respectively.^21^, ^22^ However, therapeutic strategies and drug development for *TERT* promoter mutation in HCC have been still blank. This study was aimed to solve this issue.

## 2 METHODS

### 2.1 Cell lines and regents

The THLE2, THLE3, Huh7, SK‐HEP‐1 and PLC/PRC/5 cells were got from American Type Culture Collection, HLE, HLF and Huh1 cells were got from Japanese Collection of Research Bioresources, SNU886, SNU878, SNU761 and SNU739 cells were got from Korean Cell Line Bank, and MHCC‐97H cells were got from the National Collection of Authenticated Cell Cultures (Shanghai, China). Cell lines were determined using MycoBlue Mycoplasma Detection Kit (Vazyme, D101‐01) and confirmed to be free of mycoplasma contamination. All cells were cultured in corresponding medium with 10% fetal bovine serum (FBS; Gbico). The medium were listed as follow: Dulbecco's modified Eagle's medium (DMEM) for MHCC‐97H, Huh1, Huh7, HLE and HLF; Roswell Park Memorial Institute (RPMI) 1640 for SNU761, SNU886, SNU878 and SNU739; Eagle's Minimum Essential Medium (EMEM) for SK‐HEP‐1 and PLC/PRC/5; BEGM (Lonza; CC‐3170) for THLE2 and THLE3. The PLK1 inhibitors BI2536 (S1109) and NMS‐P937 (S7255) as well as the Smad3 inhibitor SIS3 (S7959) were got from Selleck Chemicals. Blasticidin S was got from Beyotime. Puromycin and doxycline (Dox) was purchased from ThermoFisher.

### 2.2 Genomic DNA isolation and Sanger sequencing

Genomic DNA of all cell lines was extracted by Tissue DNA Kit (Omega, D3396‐02). The proximal *TERT* promoter fragment spanning G250A and G228A was PCR amplified from genomic DNA using the following primers: forward 5′–CTCCGCATGTCGCTGGTT−3′; reverse 5′—TTCACGTCCGGCATTCGTG−3′. The purified PCR product (874 bp) was subsequently sent for Sanger sequencing using the primer 5′–CTCCGCATGTCGCTGGTT−3′ to check for *TERT* promoter mutation status.

### 2.3 siRNA transfection

For siRNA treatment, HCC cells were seeded overnight to 30−50% confluency on the following day in 6‐well plates and transfected with RNA–lipid complexes containing non‐targeting siRNA, siRNAs targeting *PLK1* or *TERT* and TurboFect Transfection reagent (ThermoFisher, R0531) according to the manufacturers’ introductions. RNA‐lipid complexes containing 50 pmol of siRNA and 2.5 ul of Turbofect transfection reagent for each well. The sequences of siRNA targeting PLK1 were listed as follow: 5′–CCCUCACAGUCCUCAAUAA–3′, 5′–GAAGAUGUCCAUGGAAAUA−3′, 5′–GGUAUCAGCUCUGUGAUAA−3′. The sequences of siRNA targeting *TERT* were listed as follow: 5′–GGAGCAAGUUGCAAAGCAU−3′, 5′—UGAACUUCCCUGUAGAAGA−3′.

### 2.4 Cell proliferation assay

After seeded in 96‐well with 2000 cells per well, cells were administrated with drugs for indicated time before performing the assay with Cell Counting Kit 8 (DOJINDO, CK‐04‐500T).

### 2.5 Western blot analysis

After indicated treatment, cells were lysis and quantified by BCA. A total of 20–40 μg protein was loading. The first antibodies were incubated overnight while the second antibodies were incubated at room temperatures for 1–1.5 h. The following antibodies were used for analysis: Cell Signaling for anti‐PARP (9532), anti‐cleaved PARP (5625), anti‐caspase 3 (14220), anti‐cleaved caspase 3 (9661), anti‐Histone H3 (4499), anti‐phospho‐Histone H3 (Ser10) (53348), anti‐PLK1 (4513), anti‐Smad3 (9523), anti‐Cyclin B1 (12231), anti‐TCTP (5128), anti‐phospho‐TCTP (Ser46); Abcam for anti‐TERT (ab32020); Abmart for anti‐GAPDH (M20006M). Except anti‐GAPDH (1:5000), other antibodies were used as 1:1000 dilution. The anti‐mouse IgG, HRP‐linked antibody (Cell Signaling, 7076) or anti‐rabbit IgG, HRP‐linked antibody (Cell Signaling, 7074) was used as secondary antibodies. BeyoECL Moon (Beyotime, P0018FS) was used as staining reagent. GE AI600 was used to capture the pictures.

### 2.6 Cell cycle analysis

After indicated treatment, cells were fixed with 70% ethanol overnight, and staining by propidium iodide (PI) for 30 min. Cell arrest was detected by FACS and analysed by FlowJo V10.

### 2.7 Apoptotic experiment

Cell apoptosis was determined with Annexin V‐FITC kits (KyeGEN, KGA107). After indicated treatment, cells were treated as introductions of the kit, and measured by FACS. Finally the data were analysed by FlowJo V10.

### 2.8 Virus packaging and infection

After the confluence of 293T reached above 90% in 12‐well plate, indicated plasmid was transfected as the system (pMD2.G:psPAX2:indicated plasmid:TurboFect Transfection reagent:DMEM = 0.2 μg:0.6 μg:1.2 μg:6 μL:200 μL). The viruses were collected by filtering from 0.45 μM filter membrane after 48 h. Then, the virus infected cells with polybrene (Yeasen, 40804ES76) at final concentration of 10 μg/mL for 48 h. For HA‐tagged TERT overexpressed cell line construction, HA‐tagged TERT was cloned into pLVX‐puro by ClonExpress II One Step Cloning Kit (Vazyme, C112‐01).

### 2.9 CRISPR‐Cas9 for single‐base editing

First, SNU739 cells were infected with lentiviral pCW‐Cas9 which was inducible by Dox for Cas9 expression, and screened by 1 μg/mL puromycin for a week. Guide RNA specific to the *TERT* promoter (Top: 5′–CACCGAGGGGCTGGGAGGGCCCGGA–3′; Bottom: 5′–AAACTCCGGGCCCTCCCAGCCCCTC–3′) was cloned into lentiGuide‐mCherry‐T2A‐BSD vector. Then, SNU739 cells containing inducible Cas9 expression were infected with lentiviral gRNA‐TERT and screened with 10 μg/mL Blasticidin S for a week. Subsequently, the cells were transfected with single‐stranded HDR oligonucleotides^23^ containing the WT *TERT* promoter sequence for 6 h and induced by Dox (2 μg/mL). After 48 h, cells were sorted by FACS through mCherry expression and identified for *TERT* promoter mutation status through Sanger sequencing.

### 2.10 CRISPR‐Cas9 technology for Smad3 knockout

For knockout of Smad3, HLE cells were infected with lentiviral pCW‐Cas9 which was inducible by Dox for Cas9 expression, and screened by 1 μg/mL puromycin for a week. Guide RNA specific to the exon 2 of Smad3 (Top‐1: 5′– CACCGTGCAGGTGTCCCATCGGAAG−3′; Bottom‐1: 5′–AAACCTTCCGATGGGACACCTGCAC −3′; Top‐2: 5′–CACCGGCTCCATGGCCCGTAGCTCG −3′; Bottom‐2: 5′–AAACCGAGCTACGGGCCATGGAGCC−3′) and the exon 6 of Smad3 (Top‐1: 5′–CACCGAGAAGCGCTCCGAATTGGAG−3′; Bottom‐1: 5′–AAACCTCCAATTCGGAGCGCTTCTC−3′; Top‐2: 5′–CACCGCCAGAAGGCCGGCTCGCAGT−3′; Bottom‐2: 5′–AAACACTGCGAGCCGGCCTTCTGGC–3′) was cloned into lentiGuide‐mCherry‐T2A‐BSD vector. Then, HLE cells containing inducible Cas9 expression were infected with lentiviral gRNA‐Smad3 and screened with 10 μg/mL Blasticidin S for a week. After induced by Dox (1 μg/mL) for 48 h, single‐cell clones were sorted by FACS and identified by Western blot.

### 2.11 RNA extraction, cDNA synthesis and qRT‐PCR

Total RNA was extracted by digesting cell pellets with Trizol regent (ThermoFisher, 15596018) and isolated by chloroform, then performing RNA precipitation thereafter using isopropanol. Complementary DNA was synthesised using RevertAid First Strand cDNA Synthesis Kit (ThermoFisher, K16225) according to the manufacturer's recommendations. qRT‐PCR was performed in triplicates using PowerUp SYBR Green Master Mix (ThermoFisher, A25742). The sequences of the qPCR primers used are listed in Supplementary Table S1.

### 2.12 RNA‐seq analysis

After administrated with or without NMS‐P937 (20 or 60 nM) for 12 h, cells were digested by Trizol regent for RNA isolation and sequencing. Three biological replicates for samples were prepared. Initial isolates were checked for quality by FastQC software and filtered to remove low‐quality calls using default parameters and specifying a minimum length of 50. Processed reads were then aligned to the Homo sapiens genome assembly with Cuffmerge software. The levels of mRNA were evaluated by Fragments Per Kilo bases per Million fragments (FPKM) using Cuffquant and Cuffnorm software. The sample correlation analysis was performed using the Pearson coefficient. Cuffdiff software was used to analyse the differential expression.

### 2.13 Mouse tumour xenograft

All animal experiments were carried out in accordance with ethical guidelines and approved by Institutional Ethics Committee for Clinical Research and Animal Trials of the First Affiliated Hospital of Sun Yat‐sen University. First, cells (500 million) were injected subcutaneously into 4‐ to 5‐week‐old female Nude mice (*n* = 6 mice per group). After tumours grow about 200 mm^3^, the mice were administrated with BI2536 by tail vein injection or NMS‐P937 through the oral. At the end of experiment, mice were euthanised and their tumours were isolated for subsequent analysis.

### 2.14 Statistical analysis

All data were analysed by GraphPad Prism version 8.3. All experiments were repeated at least twice. The data were analysed by ordinary one‐way ANOVA test or t‐test as appropriate. Data are represented as mean ± SD unless otherwise noted. Statistical significance is indicated as: *p < .05, **p < .01, ***p < .001.

## 3 RESULTS

### 3.1 PLK1 inhibition selectively inhibits the growth of *TRET* promoter mutant HCC cells

**FIGURE 1:**
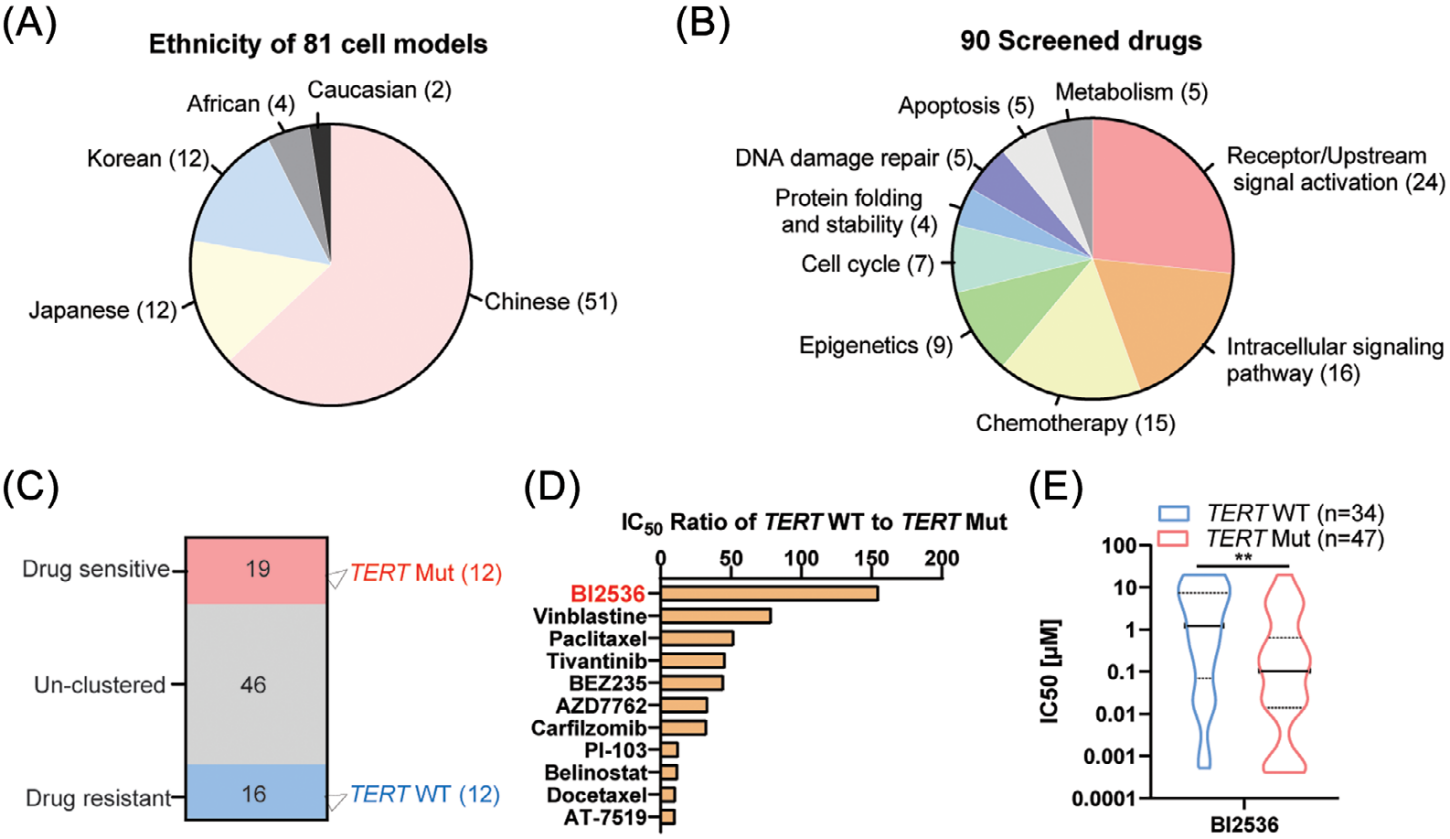
The screening schedule of drugs specially targeting to HCC cells with the mutant *TERT* promoter. (A) The ethnicity of 81 cell models. (B) The cluster of 90 screened drugs. (C) The cluster of cells according to the mutational state of TERT promoter. (D) The ranking results of IC_50_ ration of *TERT* WT to *TERT Mut*. (E) The sensitivity of all HCC cells to BI2536. ^∗*^ *p* < .01 versus *TERT* WT.

Although HCC is the disease of high incidence and mortality, there are only about 30 cell lines commercially available before 2019, which is not sufficient to capture the genomic diversity and drug screening for this disease. In 2019, Qin et al. built the Liver Cancer Model Repository (LIMORE)^24^ by collecting 31 public liver cancer cell lines and generating 50 Chinese patient‐derived models (Figure 1A). Moreover, the team performed diversified drug responses by using 90 anticancer drugs in all 81 cell models. The drugs contain 15 chemotherapeutic and 75 molecularly targeted drugs against nine cellular functions (Figure 1B), and most of these drugs were approved for clinical use (*n* = 43) or in clinical trials (*n* = 32). Base on the database, we performed the following analysis to screen the the potential drugs that selectively inhibits the growth of HCC cells with *TERT* promoter mutation (*TERT* Mut). According to the activity area of cells to the 90 clinic drugs, the cells were divided into three clusters: drug‐resistant (*n* = 16), unclustered (*n* = 46), drug‐sensitive (*n* = 19) (Figure 1C). Notably, 12 HCC cells with *TERT* wild type promoter (*TERT* WT) belong to the drug‐resistant cluster, while the drug‐sensitive cluster contains 12 HCC cells with *TERT* Mut. Subsequently, these 24 cells were selected for the following analysis. The mean values of half inhibiting concentration (IC_50_) of each drug in 12 cells of drug‐resistant cluster or drug‐sensitive cluster were calculated. The IC_50_ ratio of *TERT* WT to *TERT Mut* was ranked, and the PLK1 inhibitor BI2536 ranked at the first place (Figure 1D). Then, we enlarged the samples and confirmed that *TERT* Mut cells were more sensitive to BI2536 than *TERT* WT cells (Figure 1E).

**FIGURE 2:**
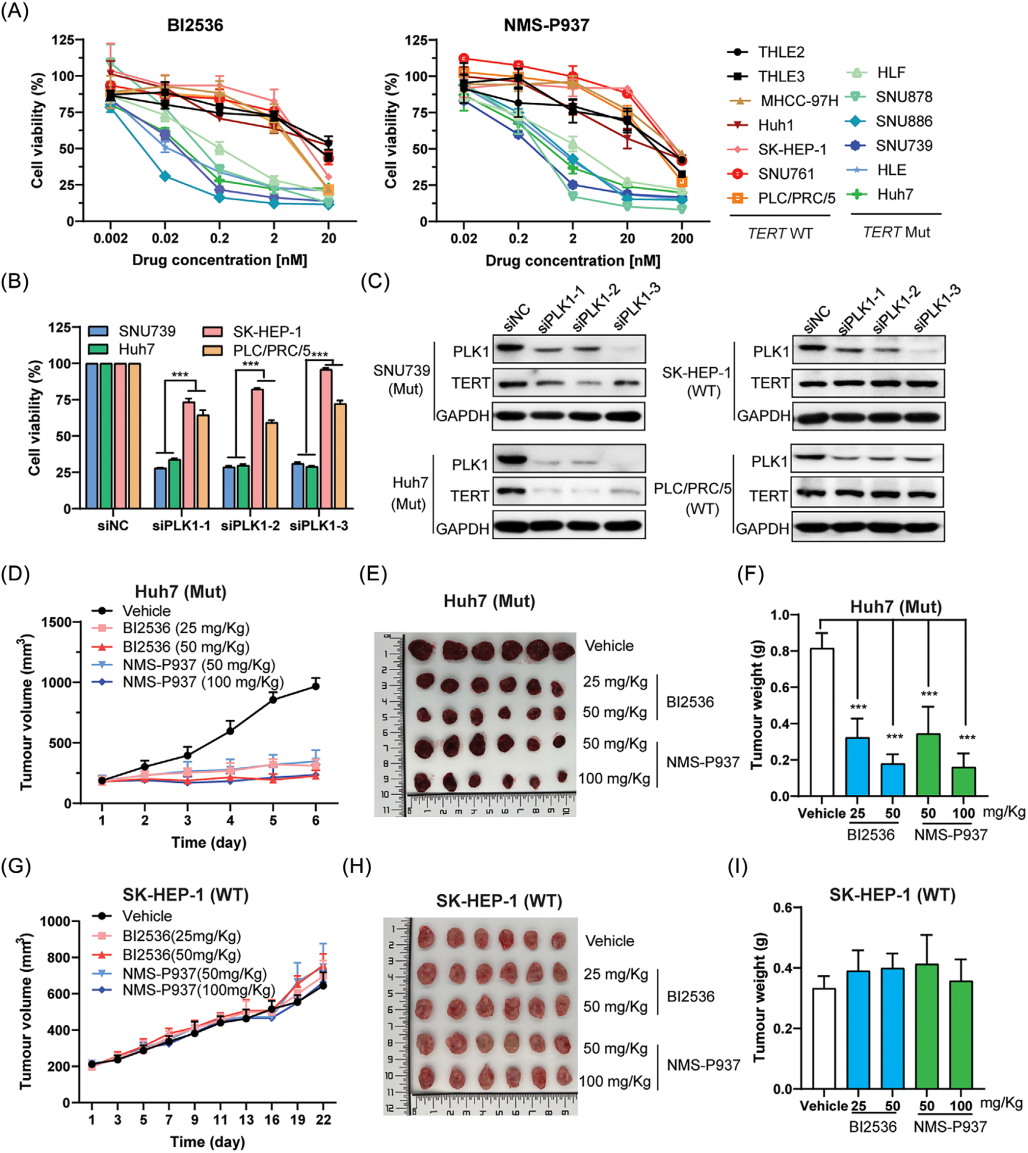
PLK1 inhibitor selectively prevents the growth of HCC cells bearing the *TERT* promoter mutation. (A) Cell viability was analysed by CCK8 assay after drug treatment for 72 h. (B) Cell viability was measured by CCK8 assay after transfection of PLK1 siRNA duplex for 72 h. ^∗∗∗^ *p* < .001, the mean value of SNU739 and Huh7 versus the mean value of SK‐HEP‐1 and PLC/PRC/5. (C) Expression of PLK1 and TERT was determined by Western blotting after transfection of PLK1 siRNA duplex for 72 h. (D) Huh7 cell‐bearing mice were treated with BI2536 by the vein injection every 2 days, twice a week or NMS‐P937 through the oral for 6 consecutive days. Plot depicting average volume of subcutaneous xenograft tumours from *n* = 6 mice per treatment group. (E) Picture shows Huh7 tumours isolated from 6 mice at the end of experiment. (F) Huh7 tumours weight was measured. ^∗∗∗^ *p* < .001 versus Vehicle. (G) SK‐HEP‐1 cells‐bearing mice were treated with BI2536 by the vein injection for 2 cycles of every 2 days, twice a week or NMS‐P937 through the oral for 10 consecutive days. Plot depicting average volume of subcutaneous xenograft tumours from *n* = 6 mice per treatment group. (H) Picture shows SK‐HEP‐1 tumours isolated from 6 mice at the end of experiment. (I) Huh7 tumours weight was measured. ^∗∗∗^ *p* < .001 versus Vehicle.

To further confirm the result, the cell viability assay was performed after BI2536 treatment in 13 cell lines with different histological and genetic background (Figure S1A), including two normal hepatocyte (THLE2 and THLE3), five HCC cells with *TERT* WT (MHCC‐97H, Huh1, SK‐HEP‐1, SNU761, PLC/PRC/5), six HCC cells with *TERT* Mut (HLF, SNU878, SNU886, SNU739, HLE, Huh7). The two hotspot mutations in TERT promoter (G228A and G250A) were detected. However, there only exits G228A mutation in all detected mutant cells and they all are heterozygous. The results showed that HCC cells with *TERT* Mut were significantly more sensitive to BI2536 than *TERT* WT (Figure 2A). Next, NMS‐P937, another PLK1 inhibitor^25^ and siRNA duplex targeting to PLK1 were used to further confirm selection of PLK1 inhibition in HCC with mutant *TERT* promoter. As shown in Figure 2A–C, the cell viabilities were significantly lower in *TERT* mutant HCC cells than wild‐type cells after PLK1 activity was inhibited or its expression were downregulated. Moreover, BI2536 and NMS‐P937 significantly inhibited the growth of Huh7 xenograft harbouring the G228A mutation (Figures 2D–F and S1B) but had little influence on the growth of SK‐HEP‐1 xenograft containing wild type of *TERT* promoter (Figures 2G–I and S1C). Together, those results showed that PLK1 inhibition selectively suppressed the growth of HCC cells harbouring *TERT* promoter mutation.

### 3.2 PLK1 inhibitors induces the apoptosis and G2/M phase arrest in HCC cells harbouring *TERT* promoter mutation

**FIGURE 3:**
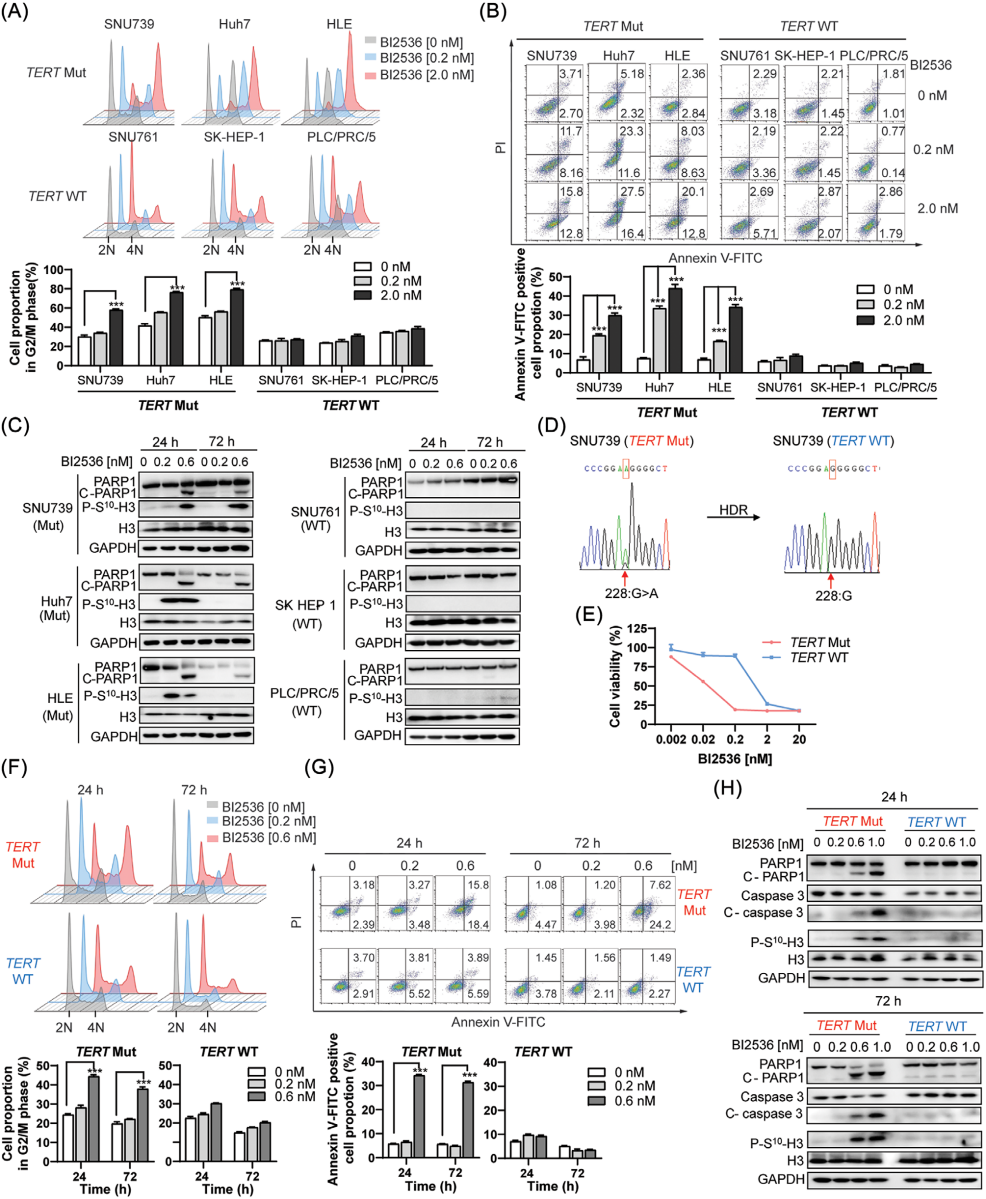
PLK1 inhibitor induces the apoptosis and G2/M phase arrest of HCC cells depending on the G228A mutation status of *TERT* promoter. (A) Cell cycle was analysed after BI2536 treatment for 24 h. ^∗∗∗^ *p* < .001 versus 0 nM. (B) Cell apoptosis was measured after BI2536 treatment for 24 h. ^∗∗∗^ *p* < .001 versus 0 nM. (C) Expression level of apoptotic and G2/M makers were measured after BI2536 treatment for 24 h or 72 h. (D) DNA sequence of SNU739 after homologous recombination by CRISPR‐Cas9 technology was shown. (E) Cell viability of SNU739 with *TERT* Mut or WT was measured after BI2536 treatment for 72 h. (F) Cell cycle arrest induced by PLK1 inhibitor in two types of SNU739 was shown. ^∗∗∗^ *p* < .001 versus 0 nM. (G) Cell apoptosis triggered by PLK1 inhibitor in two types of SNU739 cells was shown. ^∗∗∗^ *p* < .001 versus 0 nM. (H) Cell apoptotic and G2/M makers were measured in two types of SNU739 cell after BI2536 treatment for 24 h or 72 h.

To investigate how PLK1 inhibitors selectively prevents the growth of HCC cells with different *TERT* promoter, we performed cell apoptosis and cell cycle analysis. The results showed that PLK1 inhibitors specially induces the G2/M phase arrest of HCC cells carrying *TERT* mutation after 24 and 72 h treatment while wild‐type cells remained unchanged (Figures 3A, S2A and S3A). Moreover, the apoptotic status induced by PLK1 inhibitors were significantly more robust in *TERT* mutant HCC cells than wild‐type (Figures 3B, S2B and S3B). Consistent with the above results, cleaved PARP1 (the marker of apoptosis) and phosphorylated Ser10 of histone H3 (the maker of G2/M phase) were only detected in *TERT* mutant HCC cells (Figures 3C and S3C). Thus, PLK1 inhibitor specially induced apoptosis and G2/M arrest of HCC with *TERT* promoter mutation.

Herein, there existed a question whether the selective inhibition of PLK1 inhibitors was associated with the various effects of them on PLK1 activity in different HCC cells. To answer it, we examined the effect of both BI2536 and NMS‐P937 on PLK1 activity, which is marked by the level of Ser46 phosphorylation of translational controlled tumour protein (TCTP) and cyclin B1.^26^, ^27^ The results showed that phospho‐TCTP‐Ser46 were significantly decreased and cyclin B1 were obviously upregulated after treatment with BI2536 or NMS‐P937 for 3 h in HCC cells whether their *TERT* promoter is mutated or not (Figures S2C and S3D). Those results suggested that PLK1 inhibitor had similar effects on PLK1 activity in all HCC cells but play a different role in HCC cells with various *TERT* promoter.

To confirm whether the different effects of PLK1 inhibitor on HCC cells were determined by *TERT* promoter mutation status, CRISPR‐Cas9 technology was used to perform single‐base editing.^23^ After the G228A mutation was res‐covered to wild type in SNU739 cells, the effects of PLK1 inhibitors BI2536 and NMS‐P937 on cell viability, apoptosis and cell cycle of mutant cells were abolished (Figures 3D–H and S2D–G). Moreover, PLK1 inhibitor caused the similar changes of phospho‐TCTP‐Ser46 and Cyclin B1 in both types of SNU739 cells (Figure S2H). Taken together, these findings suggested that PLK1 inhibitor ‐induced apoptosis and G2/M phase arrest of HCC cells was depended on *TERT* promoter mutation.

### 3.3 PLK1 inhibitor decreases TERT expression at translational level

**FIGURE 4:**
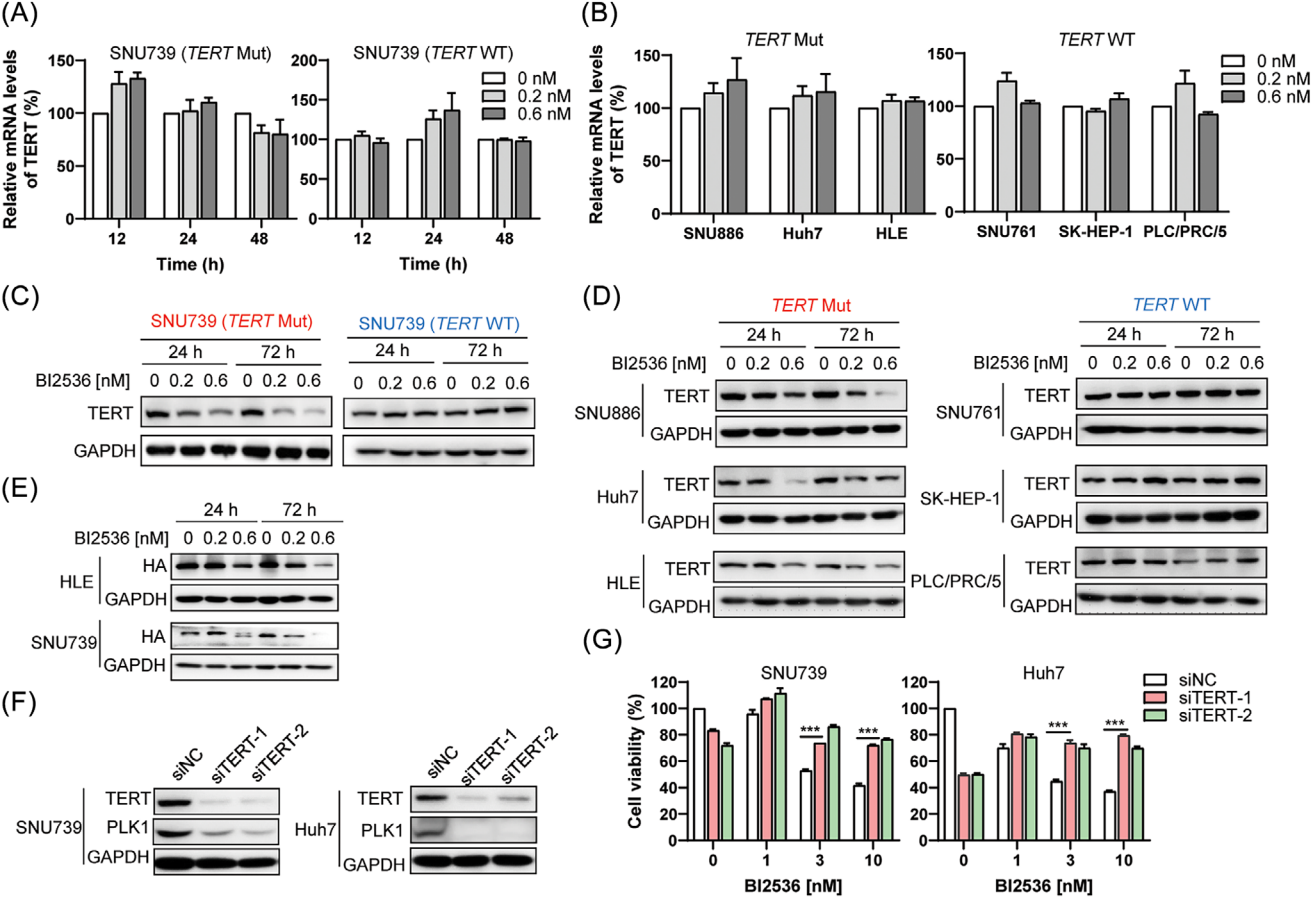
PLK1 inhibitor causes a decrease of TERT protein level but makes no difference on its transcription. (A) The transcription level of TERT in two types of SNU739 cells was assayed after BI2536 treatment for 12, 24 and 48 h. (B) The transcription level of TERT in HCC cells was measured after BI2536 treatment for 24 h. (C) Expression level of TERT in both types of SNU739 cells after BI2536 treatment for 24 or 72 h was measured. (D) Expression level of TERT in various HCC cells after BI2536 treatment for 24 or 72 h was measured. (E) Expression level of HA was measured after BI2536 treatment for 24 and 72 h. (F) Expression level of TERT was measured after transfection of TERT siRNA duplex for 72 h. (G) Cell viability was measured after BI2536 treatment for 24 h while cells were transfected by TERT siRNA duplex for 72 h. ^∗∗∗^ *p* < .001 versus siNC.

Previous studies^12^ showed that *TERT* promoter mutation created a new binding site for GABPA for *TERT* reactivation. Thus, we hypothesised that PLK1 inhibitors might decrease the expression of GABPA to selectively suppress the proliferation of HCC cells containing *TERT* promoter mutation. Unexpectedly, after detecting the transcriptional levels of *TERT* and the expression levels of GABPA (Figures 4A and B and S4A), we found that PLK1 inhibitor did not affect the transcriptional levels of *TERT* (Figure 4A and B) and had little effect on the expression of GABPA in all HCC cells (Figure S4A). Thus, transcription regulation of TERT was not involved in the growth inhibition of *TERT* mutant HCC cells induced by PLK1 inhibitors.

Next, we detected the protein levels of TERT after PLK1 inhibitors treatment. The result showed that TERT was remarkably downregulated by PLK1 inhibitor BI2536 in HCC cells carrying *TERT* mutation, while it had little effect on TERT protein level in wild type cells (Figure 4C and D). To further confirm that downregulation of TERT caused by PLK1 inhibitors was dependent on translation rather than transcription, we constructed cell lines of stable expressing TERT with HA tag in TERT mutant SNU739 and HLE cells, and treated them with BI2536. The results showed that HA which is independent of endogenous *TERT* promoter regulation was remarkably decreased (Figure 4E). Thus, PLK1 inhibitors decreased the expression of TERT on translational regulation.

Subsequently, we explored the role of TERT expression in the growth inhibition of *TERT* mutant HCC cells induced by PLK1 inhibitors. Thus, *TERT* mutant SNU739 and Huh7 cells were transfected by siRNA duplex targeting to TERT for 72 h and administrated with BI2536 for another 24 h. The results indicated that the effects of PLK1 inhibitor on *TERT* mutant HCC cells were significantly diminished when TERT was knocked down (Figure 4F and G). Moreover, the levels of PLK1 were also downregulated along with the decrease of TERT, implying the mutual regulation between PLK1 and TERT (Figure 4F). That is, PLK1 inhibitor suppressed the activities of PLK1 and downregulated the protein level of TERT, which in turn downregulate the expression of PLK1. Furthermore, PLK1 inhibitors only regulated the translational level of TERT. We were wonder whether PLK1 regulated TERT by directly interacting with TERT. Therefore, we performed co‐immunoprecipitation and immunofluorescence experiments to investigate the interaction between PLK1 and TERT. The results showed that they interacted with each other (Figure S4B). However, the results of immunofluorescence showed that they were not co‐localised in G2/M phase (Figure S4C). Thus, there exist a special signal pathway in mutant cells to respond to PLK1 inhibitor for TERT regulation.

### 3.4 Smad3 mediates the effect of PLK1 on *TERT* promoter mutant HCC cells

**FIGURE 5:**
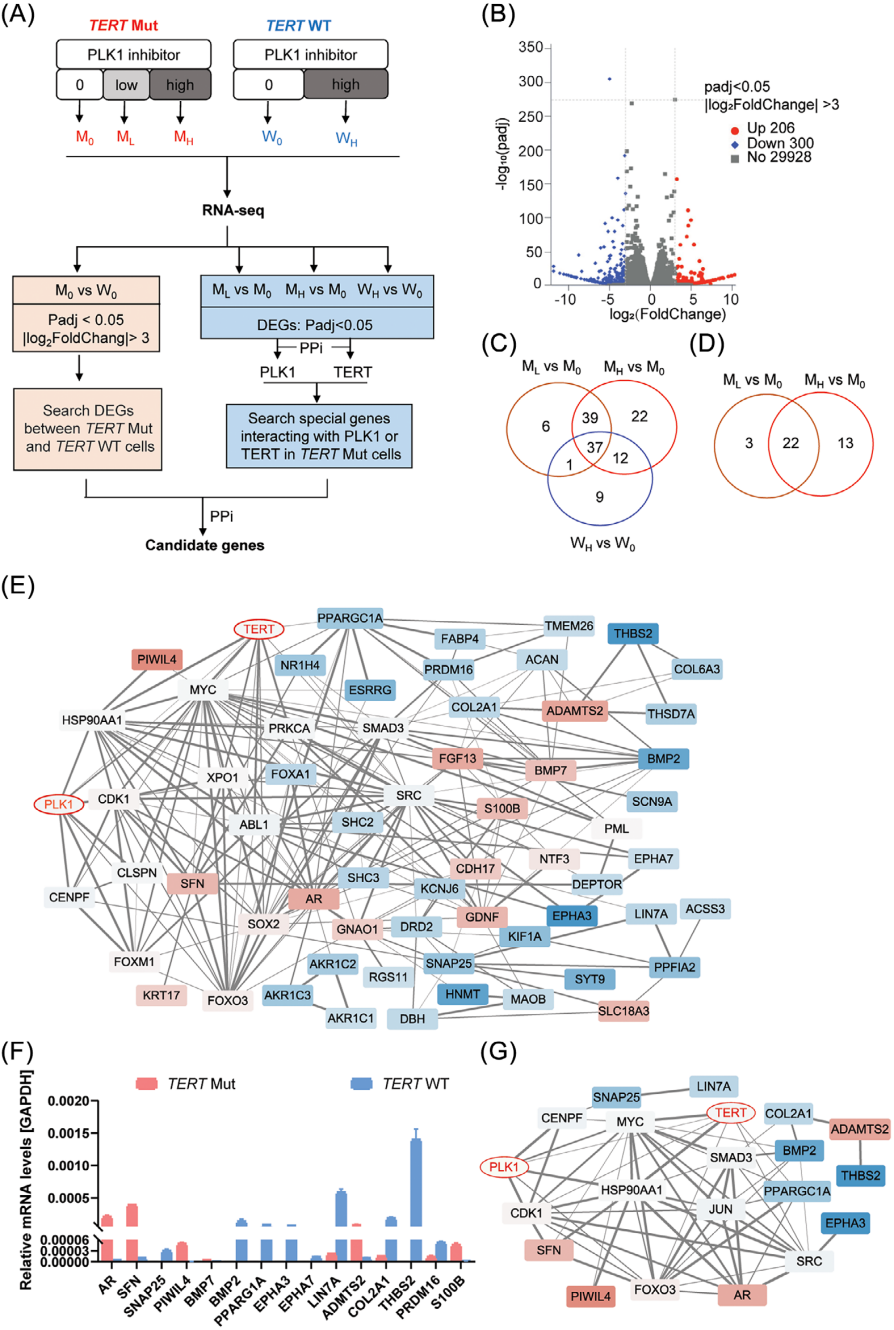
Specific signal pathway is activated by PLK1 inhibitor in SNU739 carrying the *TERT* promoter mutation. (A) Analysis strategy of RNA‐seq data was shown. (B) Differential genes between mutation and wild type of SNU739 cells were shown. (C) Venn analysis of differential gene binding to PLK1 was shown. (D) Venn analysis of differential gene binding to TERT was shown. (E) Protein‐protein interaction network of (B), (C) and (D) was structured. Red colour indicated that genes were upregulated in the mutant cells while blue colour indicated that genes were downregulated in the mutant cells. The shade of colour represented the size of different multiple. Grey colour indicated the transcription of genes was not different between the mutant cells and wild‐type. (F) The mRNA levels of target genes were assayed. (G) Protein‐protein interaction network of target genes, PLK1 and TERT was shown.

To explore the specific signal pathway in *TERT* mutant HCC cells in response to PLK1 inhibitor for TERT regulation, we made the following hypothesis: First, *TERT* promoter mutation induced differential expression of some genes; Secondly, PLK1 inhibitor selectively influenced the change of these genes in TERT mutant HCC cells; Thirdly, these special genes may directly interact with PLK1 or PLK1 downstream targets as well as TERT or its upstream regulators, since PLK1 is a kinase and TERT was regulated at translational level. Thus, we performed RNA‐sequencing and protein‐protein interaction (PPi) analysis in genetic edited SNU739 cells as well as the parent cells administrated with or without NMS‐P937 to identify key mediators for TERT regulation (Figure 5A). Briefly, mutant SNU739 cells were divided into three groups: vehicle (M_0_), low‐dose (M_L_) and high‐dose (M_H_). And wild‐type SNU739 cells were divided into two groups: vehicle (W_0_) and high‐dose (W_H_) wherein the dose of W_H_ is the same to M_H_. Based on differentially expressed genes (DEGs) analysis, 206 genes were upregulated and 300 genes were downregulated in M_0_ group of mutant SNU739 cells when comparing with W_0_ group of wild type (Figure 5B). There were 39 DEGs caused by PLK1 inhibitor interacted with PLK1 only in the mutant SNU739 cells (Figure 5C). Moreover, there were no DEG caused by PLK1 inhibitor interacting with TERT in the wild‐type SNU739 cells (Figure 5D). Herein, we proposed the hypothesis that PLK1 inhibitor may inhibit the same downstream proteins that activate different signal for regulation of TERT or different downstream targets for TERT regulation. Thus, we performed the PPi network by using DEGs in M_0_ and W_0_ group, 39 common DEGs binding to PLK1 in both M_L_ and M_H_ groups, 37 common DEGs binding to PLK1 in M_L_, M_H_ and W_H_ groups, and 22 common DEGs binding to TERT in both M_L_ and M_H_ groups. After excluding the genes that bind to TERT or PLK1 but did not bind to DEGs in M_0_ and W_0_ group, the PPi network was shown as Figure 5E. Moreover, the relative levels of these DEGs in the two cell lines were all confirmed by qRT‐PCR except for few undetectable genes (Figure 5F). Finally, these genes and the related protein that interact with PLK1 or TERT were used to build a new PPi network for final screening (Figure 5G).

**FIGURE 6:**
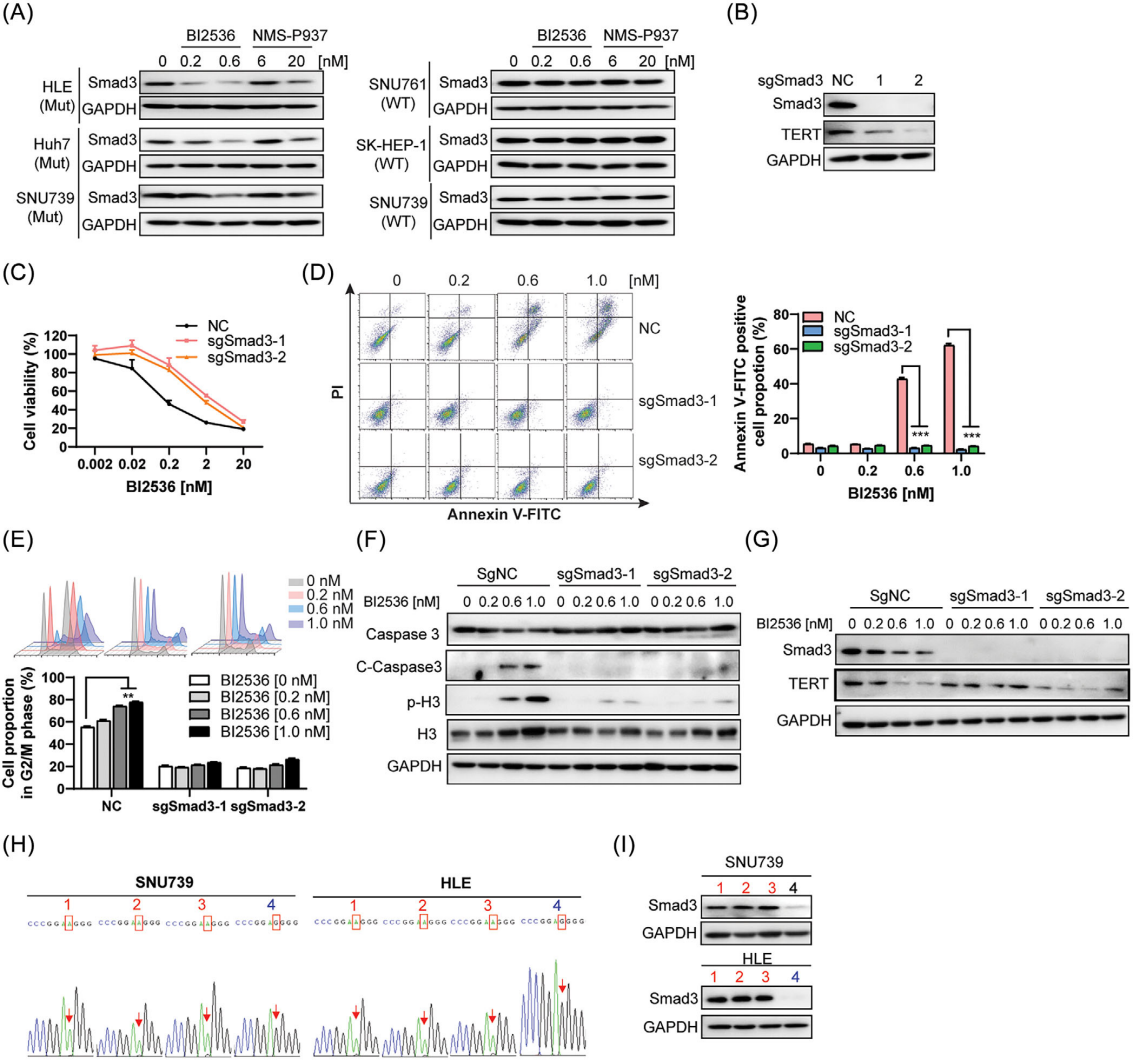
Smad3 signal is the key point in the selective inhibition of PLK1 inhibitor in HCC cells carrying distinct *TERT* promoter mutation status. (A) The expression levels of Smad3 were detected after PLK1 inhibitors treatment for 24 h. (B) The expression of Smad3 and TERT in HLE cells were detected after Smad3 was knocked out by CRISPR‐Cas9 technology. (C) Cell viability was measured after BI2536 treatment for 72 h. (D) Cell apoptosis in HLE cells was analysed after BI2536 treatment for 72 h. (E) Cell cycle in HLE cells was analysed after BI2536 treatment for 72 h. (F) The markers of apoptosis and G2/M phase in HLE cells were detected after BI2536 treatment for 72 h. (G) The levels of TERT and Smad3 were detected after BI2536 treatment for 72 h. (H) DNA sequence of SNU739 and HLE after homologous recombination by CRISPR‐Cas9 technology was shown. (I) The expression of Smad3 in cells harbouring distinct TERT promoter mutation status was detected.

As one of the key effector in the network, Myc regulated *TERT* at transcriptional level.^28^ However, the transcriptional level of *TERT* was not changed by PLK1 inhibitors (Figure 4A and B). Thus, we first detected the effects of PLK1 inhibitor on the key nodes AR which was significantly upregulated in *TERT* mutant cells and Smad3 which was centred in downregulated genes in *TERT* WT cells (Figure 5G). Comparing with SNU739 cells containing *TERT* WT, the level of androgen receptor (AR) in SNU739 cells containing the *TERT* mutation was higher and decreased by PLK1 inhibitor BI2536 or NMS‐P937 (Figure S5A). However, the basal levels of AR were undetectable in *TERT* mutant HLE and Huh7 cell lines (Figure S5B), implying that AR was not the key for response of HCC cells to PLK1 inhibitor. In contrast, Smad3 was expressed in all the cells we tested and PLK1 inhibitor treatment induced an obvious decrease of Smad3, but made no visible effect in cells containing *TERT* WT (Figure 6A). When Smad3 was knocked out by CRISPR‐Cas 9 technology (Figure 6B), the effect of PLK1 inhibitor on growth, apoptosis and G2/M arrest of TERT mutant cells were counteracted (Figure 6C–F). Those data implying that Smad3 was the key factor for response to PLK1 inhibitor in TERT mutant cells. Furthermore, TERT was also downregulated (Figure 6B) and the effects of PLK1 inhibitor on TERT expression were also attenuated when Smad3 was knocked out (Figure 6G), implying that PLK1 inhibitor regulated the expression of TERT via Smad3.

Next, we wonder to know why Smad3 was decreased in TERT mutant cells after PLK1 inhibitor treatment. Thus, we detected the expression levels of Smad3 in various homozygous mutation and wild‐type cells after single‐base editing. The results showed that genetic editing of TERT promoter mutation into the wild‐type nucleotide significantly decreased the protein levels of Smad3 (Figure 6H and I) but had little effects on transcriptional levels (Figure S5D), and induced resistance to the effects of PLK1 inhibitor on *TERT* mutant HCC cells (Figures 3D–H and S2D–G). Taken together, these data suggested that Smad3 is the key node for mediating the effects of PLK1 inhibitor on TERT expression and cellular responses in *TERT* mutant HCC cells.

### 3.5 Cooperative anti‐tumour effects of PLK1 and Smad3 inhibition in *TERT* mutant cells

**FIGURE 7:**
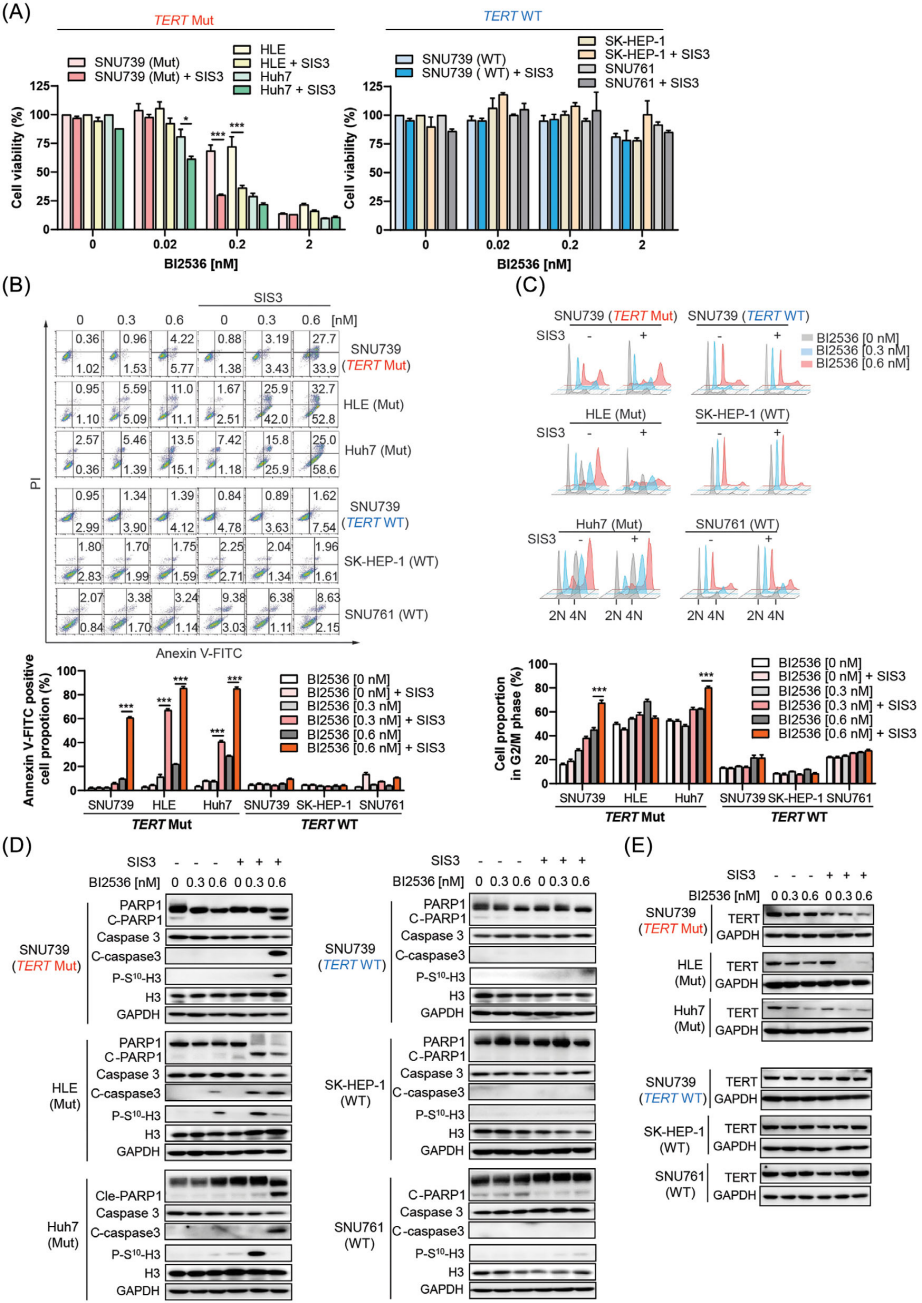
Inhibitor of Smad3 enhanced the effects of PLK1 inhibitor in *TERT* mutant cells. (A) Cell viability was measured after BI2536 treatment for 96 h when cells were pretreated with Smad3 inhibitor (5 μM SIS3) for 24 h. ^∗∗∗^ *p *< .001, cells with SIS3 versus cells without SIS3. (B) Cell apoptosis was assayed after BI2536 treatment for 96 h when cells were pretreated with Smad3 inhibitor (5 μM SIS3) for 24 h. ^∗∗∗^ *p *< .001, cells with SIS3 versus cells without SIS3. (C) Cell cycle was analysed after PLK1 inhibitor treatment for 96 h when cells were pretreated with Smad3 inhibitor (5 μM SIS3) for 24 h. ^∗∗∗^ *p *< .001, cells with SIS3 versus cells without SIS3. (D) Apoptotic and G2/M phase makers were measured after BI2536 treatment for 96 h when cells were pretreated with Smad3 inhibitor (5 μM SIS3) for 24 h. (E) Expression levels of TERT were determined after BI2536 treatment for 96 h when cells were pretreated with Smad3 inhibitor (5 μM SIS3) for 24 h.

Lastly, we assessed the effects of combined inhibition of PLK1 and Smad3 on HCC cells. As a result, we found that the Smad3 specific inhibitor, SIS3, selectively strengthened the suppression of PLK1 inhibitor BI2536 on cell viability of *TERT* mutant cells rather than wild‐type cells (Figure 7A). And the effects of PLK1 inhibitor on cell cycle and apoptosis of *TERT* mutant HCC cells were also augmented by SIS3 (Figure 7B–D). Moreover, SIS3 enhanced the downregulation of TERT induced by PLK1 inhibitor in HCC cells harbouring TERT mutation but made no change in wild‐type HCC cells (Figure 7E). Thus, these data showed a cooperative effect of PLK1 and Smad3 on TERT expression and cell growth in TERT promoter mutant HCC cells.

## 4 DISCUSSION

HCC commonly evolved from liver cirrhosis.^29^, ^30^ During the process, it involves genetic mutation, immune escapes and so on.^7^, ^31^ The therapeutical strategies and drug development were decided by the drivers, suppressors and their regulator of HCC development. In past 10 years, novel driver genes were discovered in HCC, such as mutations in the CTNNB1, TERT promoter, TP53.^8^, ^32^, ^33^ However, most of them in HCC are currently undruggable except FGF19 and Met.^34^, ^35^ TERT reactivation caused by mutation of core promoter is the most common in HCC. G228A (Mutation ID: MU832963) and G250 (Mutation ID: MU830690) are the main hotspot mutation of TERT promoter in human cancers and they both reactivates *TERT* expression by creating a de novo binding site (TTCCGG) for ETS transcription factors, but 90% mutation in HCC are G228A. Lu et al. team found it will inhibit the tumour growth in brain and liver through editing the mutated *TERT* promoter,^36^, ^37^ implying the importance and possibility of precision therapy aiming at TERT promoter mutation in HCC. To date, there are no drugs for that. Herein, we uncovered that the PLK1 inhibitors prefers to inhibit the growth of HCC cancer cells harbouring *TERT* promoter mutations (Figures 1 and 2). Importantly, the sensitivity of PLK1 inhibition to *TERT* promoter mutation was diminished after we recovered the mutant nucleotide to the wild‐type form by CRISPR‐CAS9 technology (Figures 3 and S2). These findings strongly suggested PLK1 as a novel therapeutic target for *TERT* mutant HCC. Distinct from previous drug development for HCC through targeting the telomerase activation function of TERT,^38^, ^39^, ^40^ PLK1 inhibitor is screened on the basis of the single nucleotide mutation in *TERT* promoter rather than TERT itself, which may have some potential adverse effects because of the high telomerase activity in rapidly growing cells such as germ cells, immune cells and stem cells.^41^, ^42^, ^43^

Since the hotspot mutations in *TERT* promoter had been well accepted to create de novo binding sites for GABPA/GABPB complex, most of previous studies aiming at targeting mutant *TERT* in human cancers were focused on targeting GABPA and/or GABPB directly or indirectly. Our recent study showed that FOS inhibitor T‐5224 suppressed the growth of *TERT* mutant cancer cells through downregulating GABPB and the transcription of *TERT* in the presence of *TERT* promoter mutation.^44^ Mancini et al. indicated that genetic disruption of GABPβ1L, a tetramer‐forming isoform of GABP, is dispensable for normal development but silence TERT in a *TERT* promoter mutation‐dependent manner in glioblastoma.^22^ Moreover, a recent study by Agarwal et al. reported that transcription factor TRIM28 activates the mutant *TERT* expression and revealed mTORC1‐mediated phosphorylation of TRIM28 could be a therapeutic target for *TERT* mutation in bladder cancer.^21^ It should be noted that all the above‐mentioned studies rely on the transcriptional regulation of the mutant *TERT* promoter. In contrast, our data demonstrate that PLK1 inhibition decreased the protein level of *TERT* but does not affect its transcription in *TERT* mutant HCC cells (Figure 4), indicating that there is different mechanism for the selectivity of PLK1 inhibition between mutant and wild‐type cells.

Subsequently, we found that PLK1 inhibitors induced Smad3 downregulation in *TERT* mutant cells and knockout of Smad3 sharply attenuated the effects of PLK1 inhibitor on such cells (Figure 6), demonstrating a key role of Smad3 in mediating the responses of *TERT* mutant cells to PLK1 inhibitors. Smad3 is responsible for transducing signal triggered by transforming growth factor β (TGF‐β) or bone‐morphogenetic proteins (BMPs/GDFs) from the membrane into the nucleus, thereby executing the downstream response by directly regulating gene expression.^45^, ^46^ Previous studies showed that TGF‐β‐induced Smad3 activation repressed TERT expression by directly binding to TERT gene's promoter and inhibiting its transcription in breast cancer cell line MCF‐7.^47^, ^48^ While our results showed that Smad3 positively regulated TERT expression at translational level (Figure 6B) rather than transcriptional level (Figure S5C), and manipulated the TERT promoter mutation to wild type induced decrease of Smad3. These findings suggested a cellular context dependent manner of TERT regulation by Smad3. Furthermore, we observed that *TERT* promoter mutation upregulated the expression of Smad3 at translational level (Figure 6H and I) rather than transcriptional level (Figure S5D) to respond to PLK1 inhibitors in HCC mut cells, indicating that TERT promoter mutation may not only enhance the level of *TERT* itself but also regulate the levels of other genes. Nevertheless, how *TERT* promoter mutation affect the expression of Smad3 needs to be further explored.

Finally, we found that SIS3, a specific inhibitor of Smad3, enhanced the effects of PLK1 inhibitor on the growth, apoptosis, and cell cycle in HCC cells carrying *TERT* mutation in low concentration. Furthermore, Smad3 inhibitor cooperated with PLK1 inhibitor to decrease the level of TERT in *TERT* mutant cells (Figure 7E). Thus, it may be a promising strategy for HCC therapy by combination of Smad3 and TERT inhibitors.

## 5 CONCLUSION

In conclusion, our findings demonstrated that genetical or pharmacological PLK1 inhibition selectively induced TERT downregulation, cell cycle arrest and apoptosis in HCC cells harbouring *TERT* promoter mutations through Smad3. Our findings encourage a clinical trial on PLK1 inhibitors single or combination with Smad3 inhibitors in *TERT* mutant HCC patients.

## AUTHOR CONTRIBUTIONS

ZP, RL and QT conceived and conceptualised the study; QT, GH, YS, YC, GW, MZ, MC and SL performed experiments and collected original data; QT, GH, YS, YC, RL and ZP analysed the data; QT prepared the figures and drafted the manuscript, with inputs from all authors. ZP and RL acquired funding, supervised the work and were responsible for the project administration. All the authors read and approved the final manuscript.

## CONFLICT OF INTEREST STATEMENT

The authors declare no competing interests.

## ETHICS STATEMENT

All experiments involving animals were approved by Institutional Ethics Committee for Clinical Research and Animal Trials of the First Affiliated Hospital of Sun Yat‐sen University (approval form: 2022‐012).

## Supporting information

Supporting Information

Supporting Information

Supporting Information

Supporting Information

Supporting Information

Supporting Information

## Data Availability

All the relevant data supporting the findings of this study are available from the corresponding author on reasonable request. Additional data are made available in supporting information.
