# Supplementary figures and images for: Therapeutic targeting of PLK1 in TERT promoter‐mutant hepatocellular carcinoma
Source: Clin Transl Med. 2024 May 20;14(5):e1703. doi: 10.1002/ctm2.1703 (PMC11106514; doi:10.1002/ctm2.1703)

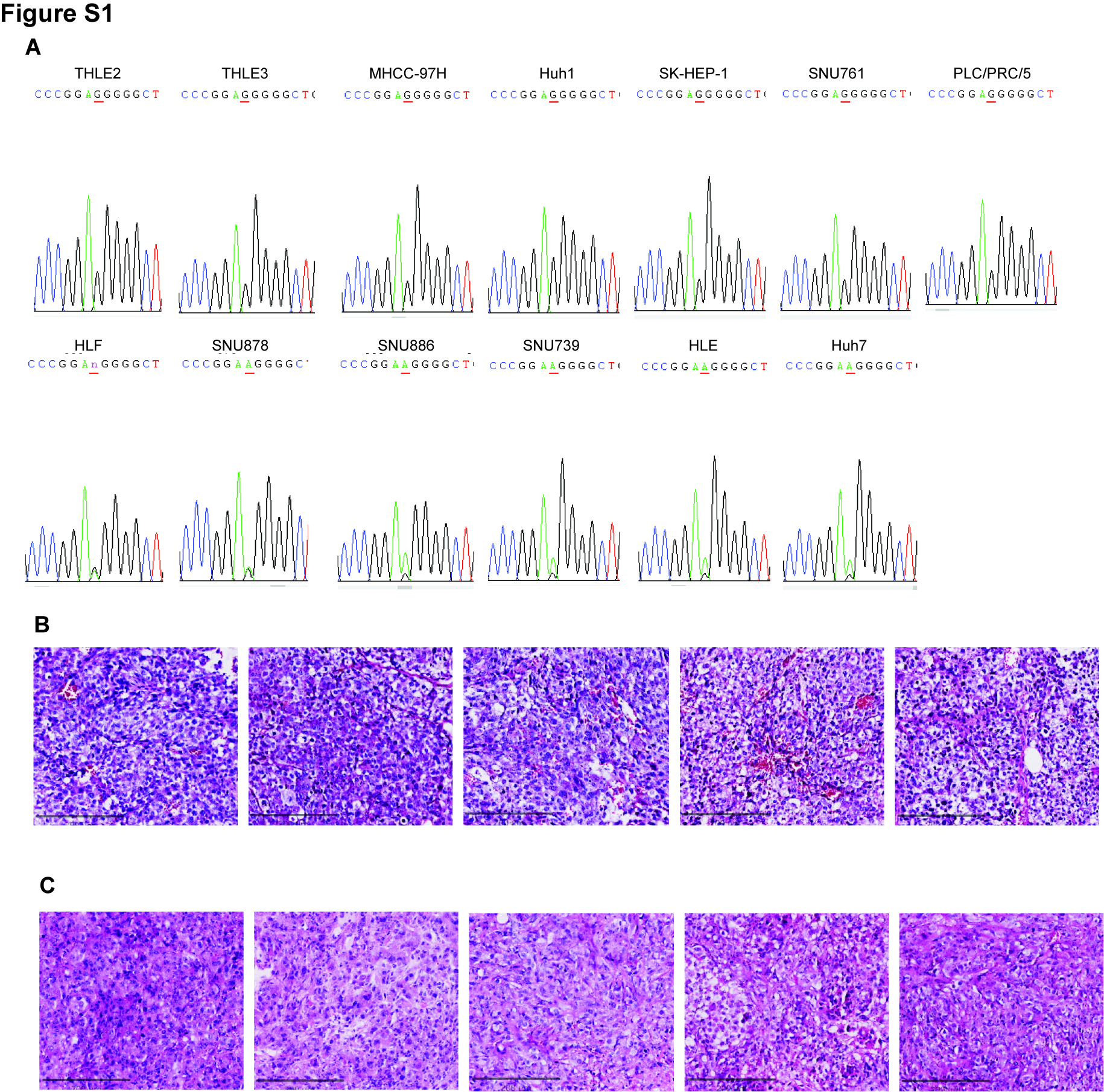

Supplement: Supplementary file 1 — Supporting Information [file CTM2-14-e1703-s005.tif]

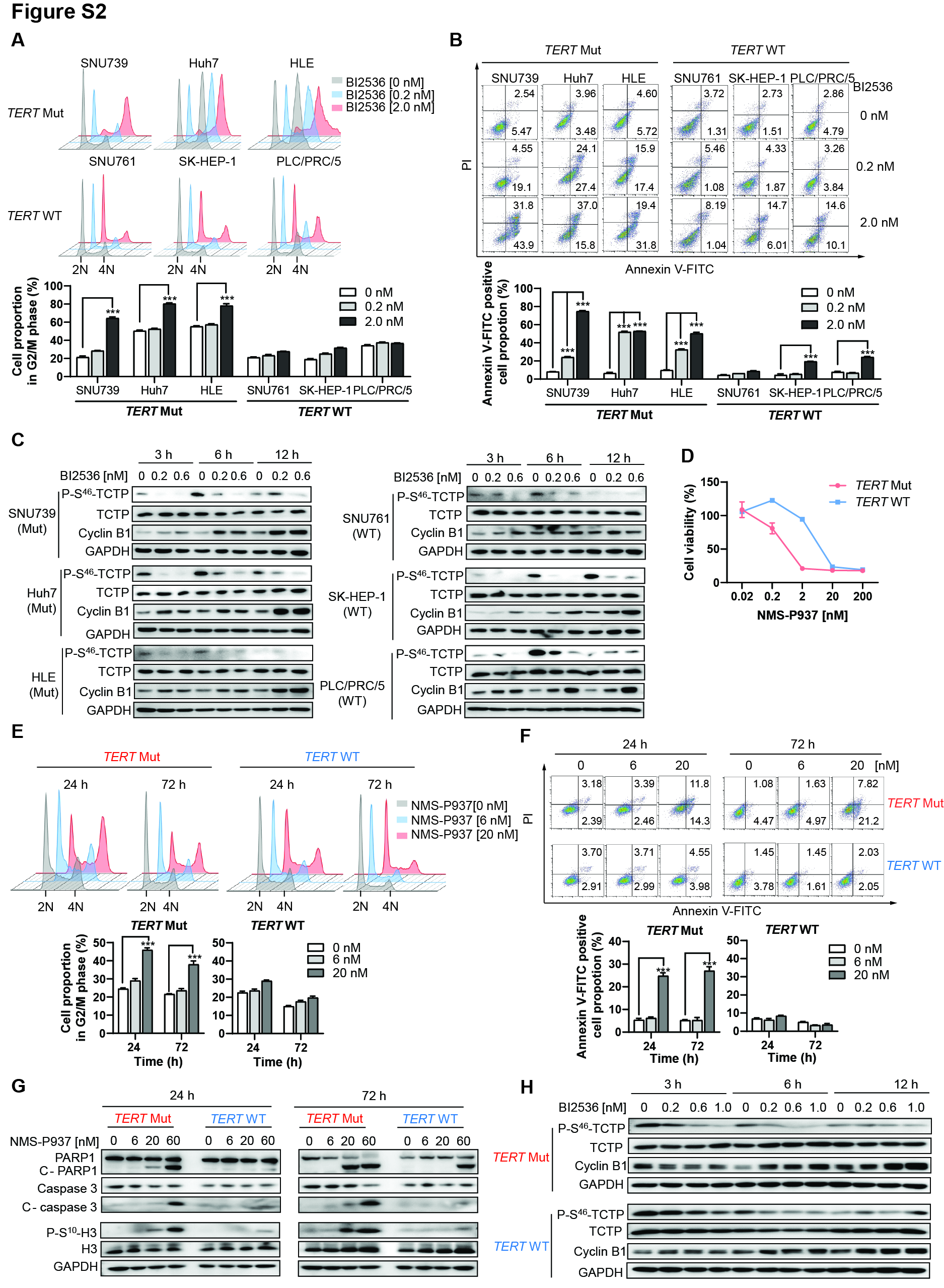

Supplement: Supplementary file 2 — Supporting Information [file CTM2-14-e1703-s004.tif]

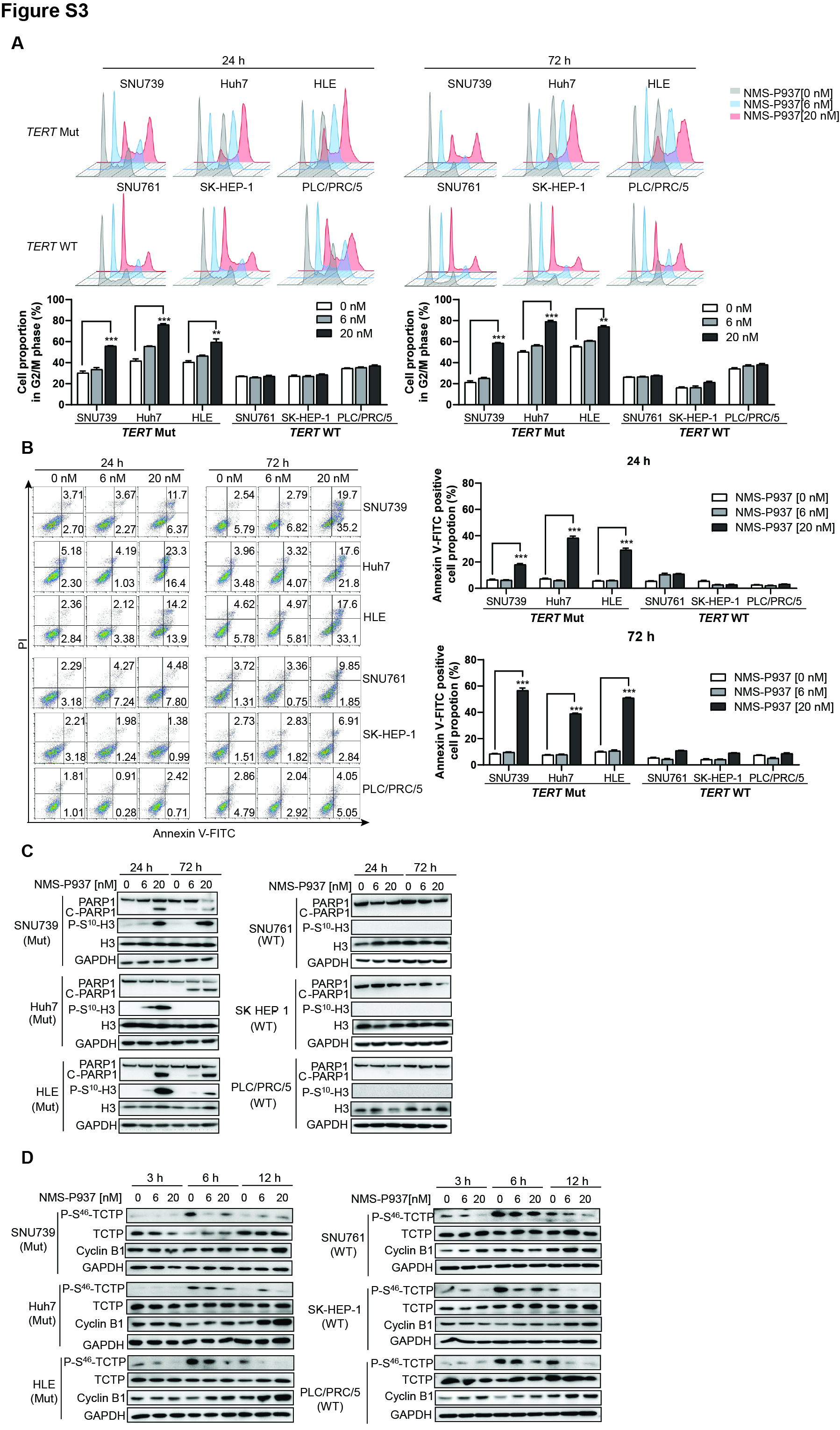

Supplement: Supplementary file 3 — Supporting Information [file CTM2-14-e1703-s002.tif]

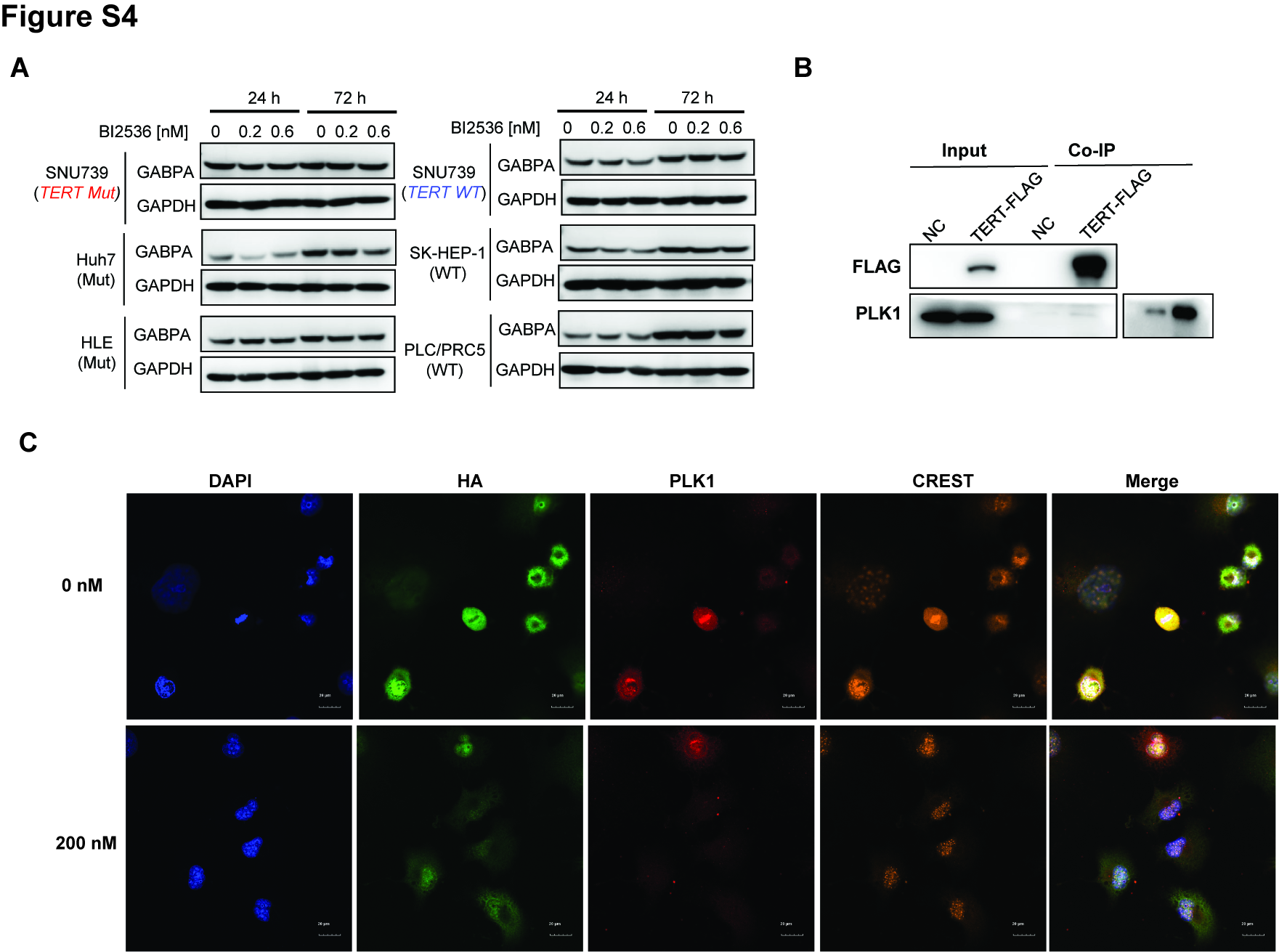

Supplement: Supplementary file 4 — Supporting Information [file CTM2-14-e1703-s001.tif]

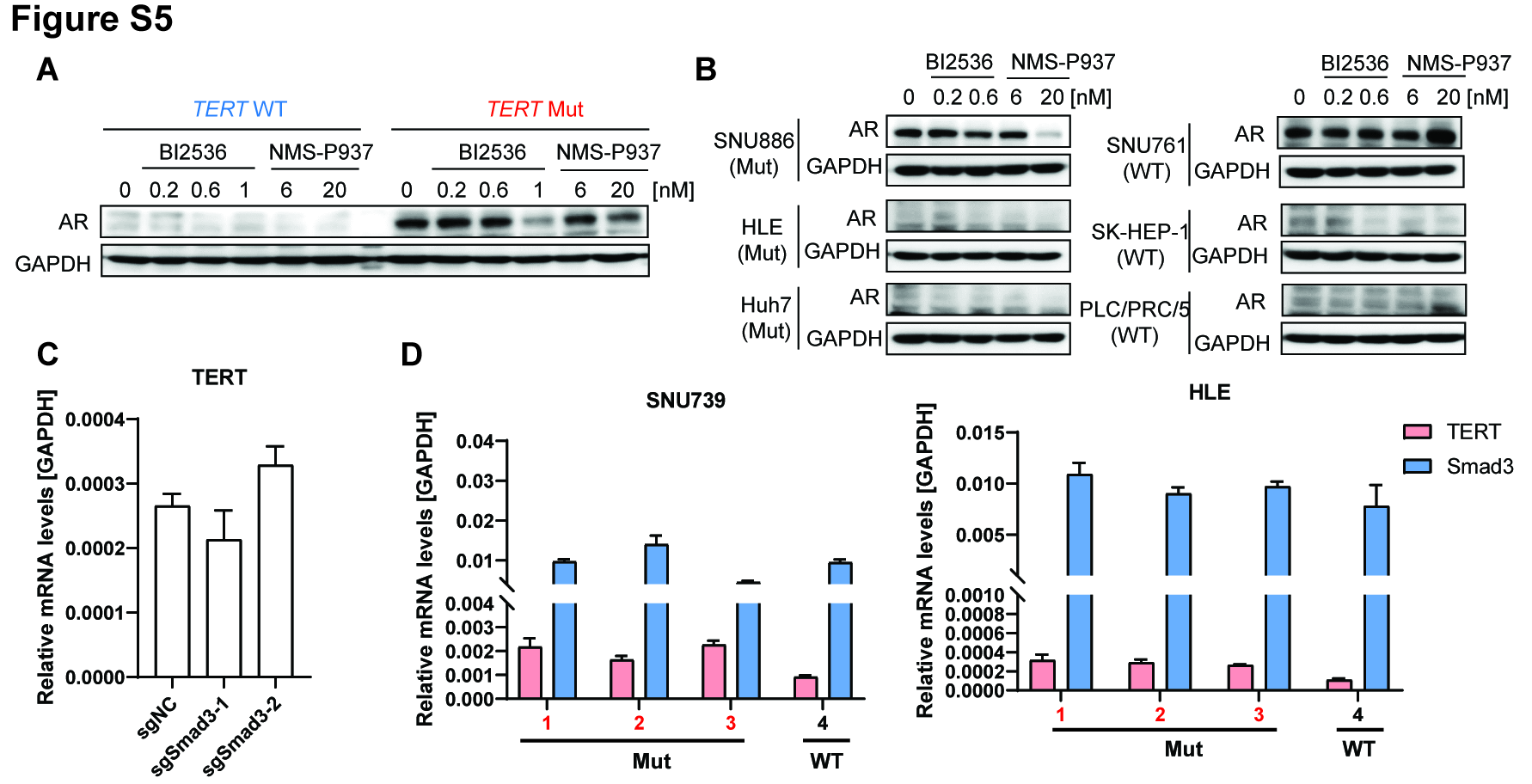

Supplement: Supplementary file 5 — Supporting Information [file CTM2-14-e1703-s003.tif]
