# Supplementary material for: Therapeutic targeting of PLK1 in TERT promoter‐mutant hepatocellular carcinoma
Source: Clin Transl Med. 2024 May 20;14(5):e1703. doi: 10.1002/ctm2.1703 (PMC11106514; doi:10.1002/ctm2.1703)
Supplement: Supplementary file 6 — Supporting Information [file CTM2-14-e1703-s006.docx]

**Methods**

**1. Hematoxylin and eosin (HE) staining**

HE staining kit was purchased from Solarbo (G1120). HE staining was performed as manufacture’s introduction. The pictures were captured by fully automatic digital pathological section scanner (Kfbio/KF-PRO-020) and analyzed by Qupath software.

**2. Co-immunoprecipitation (Co-IP)**

293T cells were seeded into 100mm dish and cultured for 24 h to a 80–90% confluence. Totally, 4 μg plvx-TERT tagged by FLAG or pNC were transfected into 293T cells, separately. After cultured for 24 h, the cells were collected. After dissolved in 550μL lysis buffer on ice for 30min and the mixture was centrifuged at 12,000rpm for 30min. Totally, 40μL supernatant was mixed with 10μL 5× loading buffer and denaturalized at 100°C for 10min, while the others were mixed with 10μL FLAG-conjugated agarose beads and gently shook overnight at 4°C. The mixture was centrifuged at 3000rpm for 3min and the pellets were washed by TBS for five times. Finally, the pellets were resuspended in 30μL 5× loading buffer, denaturalized at 100°C for 10min and detected by Western blot.

**3. Immunofluorescence**

Cells were seeded into 35 mm confocal dish for 24 h and treated with 2 mM thymidine for 18 h. Then, the medium was removed, washed with PBS and incubated with fresh medium for 9 h. Subsequently, cells were incubated with 2 mM thymidine for another 18 h. Next, cells were released by washing with PBS and incubating cells in fresh medium for 4 h to enter into G2/M phase. Then, cells were treated with vehicle or 200 nM BI2536 for 2 h. Cells were fixed with methanol pre-cooled at -20° for 5 minutes at room temperature, washed with PBS and blocked with PBS containing 1% BSA (w/v) and 0.3% Triton X‐100 (v/v) for 1 hour at room temperature. Cells were then incubated with the indicated primary antibody diluted with PBS containing 1% BSA (w/v) and 0.3% Triton X‐100 (v/v) overnight at 4 °C. Cells were washed three times with PBS and incubated with the corresponding fluorescent secondary antibody for 2 hour at room temperature. After three washes with PBS, cells were stained with 10 μg/mL DAPI for 10 minutes, washed with PBS and imaged by laser scanning confocal microscope (Olympus/FV300). Antibodies were listed as follow: anti-HA rabbit (SIGMA, H6908, 1:1000), anti-Plk1 mouse (Abcam, ab17057, 1:500), Human anti-centromere (CREST, Antibodies Inc. 15-234-0001, 1:1000).

**Table**

**Table S1 qRT-PCR primer sequences**

| Gene name | Forward primer (5’→3’) | Reverse primer (5’→3’) |
| --- | --- | --- |
| S100B | ACAAGGAAGAGGATGTCTGAGC | GCCGTCTCCATCATTGTCCA |
| PRDM16 | AGGCGGTCTGTTAGCTTTGG | GTGGAGAGGAGTGTCTTCGG |
| THBS2 | AGCACGCAAGCTGGTCAC | CTCCTTCTGCCGCATGATCT |
| SCN9A | CCGTTTCAATGCCACACCTG | TGACGACAAAATCCAGCCAGT |
| COL6A3 | ACGCAGTGAGTGGGAAAAGT | TATATCAGCAGCCGCACCATT |
| THSD7A | ACGTTCCAAGAGCGGAGAAA | TCCACAGATAGAGGGTGGGC |
| COL2A1 | ATGAGGGCGCGGTAGAGAC | TCACAGACACAGATCCGGCA |
| FGF13 | AGATGCGAGGCCATTACCTG | GCCCTGTTGCCCTTCTGAT |
| ADAMTS2 | CAGGGCCGCTTGGTGTC | GACGTAGAGACAGCTCCCGA |
| LIN7A | TGCACAAGCTACAATCCCTCA | TGGCCTTCACTAGCTGCAAA |
| SYT9 | GCTGGCGAAGAGCTGCAT | GAAATCCTGGCAGCTGTCGT |
| KIF1A | CACTGACACCAACACTGTGC | AGCTTCTCCTCCCAGGTCTC |
| PPFIA2 | TGGTGCCATCATGTCTGCTT | AGCTTCTCCTCCCAGGTCTC |
| EPHA7 | TTCCAGGCACCAAAACCTACA | TCAAACTGCCCCATGATGCT |
| EPHA3 | GCACAACAGGTGACTGGCTT | ACTTCCGTCCCGTGCTTTAC |
| PPARGC1A | GTTGCCTGCATGAGTGTGTG | TCACTGCACCACTTGAGTCC |
| BMP2 | CTGCGGTCTCCTAAAGGTCG | GGGGTGGGTCTCTGTTTCAG |
| BMP7 | GGGCTTCTCCTACCCCTACA | ACGTCTCATTGTCGAAGCGT |
| PIWIL4 | TGCTTGCTCGGACCTTGAAT | CCATCACGTCCTTGCTGAGT |
| SNAP25 | ACGCATTGAGGAAGGGATGG | GTTCGTCCACTACACGAGCA |
| SFN | ACTACGAGATCGCCAACAGC | ACAGTGTCAGGTTGTCTCGC |
| AR | GACATGCGTTTGGAGACTGC | GTTTCTTCAGCTTCCGGGCT |
| GAPDH | AAGAAGGTGGTGAAGCAGG | TTGACAAAGTGGTCGTTGAG |

**Figure legends**

**Figure S1 DNA sequencing of *TERT* promoter region in HCC cells and HE staining**. (**A**) DNA sequencing of *TERT* promoter region in HCC cells. The red line marks the mutant site. (**B**) HE staining of Huh7 xenograft. (**C**) HE staining of SK-HEP-1 xenograft.

**Figure S2** **PLK1 inhibitor induces the apoptosis and G2/M phase arrest of HCC cells depending on the C228T mutation status of TERT promoter**. **(A)** Cell cycle was analyzed after BI2536 treatment for 72 h. ^∗∗∗^ *p* <0.001 versus 0 nM. **(B)** Cell apoptosis was measured after BI2536 treatment for 72 h. ^∗∗∗^ *p* <0.001 versus 0 nM. **(C)** The expression levels of PLK1 downstream proteins in two types of HCC cells were measured after BI2536 treatment for 3 h, 6 h and 12 h. **(D)** Cell viability of SNU739 with *TERT* Mut or WT was measured after NMS-P937 treatment for 72 h. **(E)** Cell cycle arrest induced by NMS-P937 in two types of SNU739 cells was shown. ^∗∗∗^ *p* <0.001 versus 0 nM. **(F)** Cell apoptosis induced by NMS-P937 in two types of SNU739 cells was shown. ^∗∗∗^ *p* <0.001 versus 0 nM. **(G)** Cell apoptotic and G2/M makers were measured in two types of SNU739 cell. (H) The expression levels of PLK1 downstream proteins in two types of SNU739 cells were measured after BI2536 treatment for 3 h, 6 h and 12 h.

**Figure S3 NMS-P937 induces the apoptosis and G2/M phase arrest of HCC cells with *TERT* mutation**. **(A)** Cell cycle was analyzed after NMS-P937 treatment for 24 or 72 h^∗∗∗^ *p* <0.001 versus 0 nM. **(B)** Cell apoptosis was measured after NMS-P937 treatment for 24 or 72 h. ^∗∗∗^ *p* <0.001 versus 0 nM. (**C**) Cell apoptotic and G2/M makers were measured after NMS-P937 treatment for 24 or 72 h. **(D)** The expression levels of PLK1 downstream proteins in two types of HCC cells were measured after NMS-P937 treatment for 3 h, 6 h and 12 h.

**Figure S4 The effects of PLK1 inhibitor on GABPA and the interaction between PLK1 and TERT**. **(A)** The levels of GABPA were detected after BI2536 treatment for 24 h and 72 h**. (B)** The interaction between PLK1 and TERT. **(C)** The co-localization of PLK1 and TERT.

**Figure S5 The effects of PLK1 inhibitor on regulation of AR and transcriptional levels of TERT and Smad3**. **(A)** The levels of AR in mutant or wild-type SNU739 cells was detected after PLK1 inhibitor treatment for 24 h. **(B)** The levels of AR in mutant or wild-type HCCs was detected after PLK1 inhibitor treatment for 24 h. (**C**) The transcriptional levels of TERT in Smad3 knock-out cells of HLE. **(D)** The transcriptional levels of TERT and Smad3 in mutant and wild-type cell.
